# Secondary headaches in pregnancy and the puerperium

**DOI:** 10.3389/fneur.2023.1239078

**Published:** 2023-09-28

**Authors:** Suzan Khoromi

**Affiliations:** University of California, San Diego, La Jolla, CA, United States

**Keywords:** acute headache, pregnancy, postpartum headache, puerperium, secondary headache

## Abstract

Headache during pregnancy can be due to primary causes such as migraine but can also be a presenting symptom of secondary causes including life threatening conditions. This is a minireview of secondary causes of headache during pregnancy and the puerperium. Unique alterations in physiological and vascular functions as well as in the coagulation pathway which occur during pregnancy increase the risk of most of these secondary conditions which include preeclampsia, eclampsia, hemorrhagic stroke, cerebral venous, sinus thrombosis, reversible cerebral vascular syndrome, and posterior reversible encephalopathy. Marked increase in progesterone level in pregnancy is also associated with the growth of tumors such as meningiomas, as 70% of these tumors are positive for progesterone receptors and increase in size can lead to headache along with other neurological symptoms. Hemodynamic changes can lead to the growth of meningiomas as well. Although hormone producing pituitary tumors are usually not conducing to pregnancy, women with known pituitary tumors who do get pregnant may become symptomatic during pregnancy and develop secondary headache. Another rare cause of secondary headache during pregnancy is pituitary apoplexy. Although its occurrence is uncommon, it needs to be properly recognized and treated to avoid endocrine and visual complications. Other rare entities with increased incidence during the puerperium such postdural puncture headache will be also discussed. In summary, new onset headache during pregnancy deserves special attention because in the absence of proper recognition and treatment, secondary headache disorders can endanger the life of the mother and the fetus.

## Introduction

This is a mini review of secondary causes of headaches during pregnancy and the puerperium. Hemodynamic, endocrine and physiological changes occur during pregnancy that put women at risk for neurological syndromes from cerebral vascular complications as such as hemorrhagic stroke to the growth of tumors such as meningiomas. Because headache in pregnancy can be the presenting symptom of these and other ominous syndromes that could not only be life threatening to the mother but also be a danger to the fetus it is an important to recognize and treat these conditions appropriately.

### Studies of headache during pregnancy

There is a paucity of studies focusing on acute headache in pregnant and postpartum women.

Ramchandren et al. reported retrospectively on 63 pregnant women presenting to the ED with headache or other neurological symptom (1). In this study 43% of patients presenting with headache had abnormal neurological examination and 27% were found to have neuroimaging findings consistent with secondary headache **(SH)** including cerebral venous thrombosis (CVT) in 6%, reversible cerebral vasoconstrictive syndrome (RCVS) in 6%, intracranial hemorrhage (IH) resulting from posterior pituitary bleed or ruptured aneurysm in 3% and sinusitis in 8% of patients, respectively (Table 1). The odds of an abnormal neuroimaging finding was 2.7 times higher in patients with an abnormal neurological examination. None of the demographic or clinical variables included in this study were found to be predictive of intracranial pathology.

**Table 1:** Comparative frequencies of secondary headaches.

Ramchandren et al. (1)	Raffaelli et al. (4)	Raffaelli et al. (2)	Robbins et al. (3)
CVT (6%)	Viral infections (17.2%)	ICH (28.6%)	Preeclampsia (13.6%)
RCVS (6%)	Sinusitis (12.5%)	CVT (23.8%)	PRES (4.3%)
IIH (3%)	Preeclampsia (9.4%)	Acute stroke (14.3%)	Eclampsia (2.9%)
ICH (3%)	PRES (6.3%)	PRES (19%)	ICH (1.4%), CVT (1.4%)
Sinusitis (8%)	HELLP (4.7%)	Sinusitis (14.3%)	Pituitary apoplexy (3.6%)

CVT, cerebral venous thrombosis; RCVS, reversible cerebral vasoconstriction syndrome; IIH, idiopathic intracranial hypertension; ICH, intracranial hemorrhage; PRES, posterior reversible encephalopathy syndrome; HELLP, hemolysis, elevated liver enzymes, and low platelet count.

Robbins et al. reported on 142 pregnant women presenting with an acute severe headache to the ED (3): 35% of patients had secondary headache disorders. Headache was attributable to pre-eclampsia in 13.6%, Posterior reversible encephalopathy syndrome in 4.3%, eclampsia in 2.9%, HELLP in 1.4%; acute arterial hypertension in 0.7%, RCVS in 0.7%.

Other causes of SH included pituitary adenoma/apoplexy in 3.6% of patients, CVT in 1.4% and IH in 1.4% (Table 1). Sixty seven percent of patients with SH presented with new onset headache in their third trimester. Non-gestational hypertension was the most significant risk factor for SH (12.2% of patients with SH vs. 1.1% of patients with primary headaches). Relative odds of SH were significantly higher in patients with a lack of pregestational headache history and patients with elevated blood pressure (BP) during pregnancy. SH was not associated with any characteristic features. One limitation of this study was 40.8% of patients were African American and 40.8% were Hispanic whereas only 8.2% were Caucasian.

Raffaelli et al. did a retrospective cross-sectional analysis of 151 pregnant women who underwent a neurological consultation due to acute headache (4). Forty two percent of these patients had secondary causes of headache. The main causes of SH in this study were: hypertensive disorders of pregnancy in 22% of the patients, including preeclampsia in 9.4%, PRES in 6.3% and HELLP syndrome in 4.7%, respectively. Nonvascular causes of secondary headache included: common viral infection in 17.2%, acute sinusitis in 12.5% (Table 1). In women with SH, elevated blood pressure was present among 31.7%, and abnormal neurological findings among 35.9%. Progressive pain dynamics in both the first and third trimester were significantly associated with a SH diagnosis (*p* = 0.037 and *p* = 0.014 respectively). Subjective pain of greater or equal to 8/10 was significantly associated with secondary headaches in the first trimester (*p*-0.04). Laboratory values that correlated with SH included abnormal CRP, abnormal platelet count and abnormal liver function.

In a separate study, the same group analyzed the secondary causes of headache in the subset of the same 151 patients who had undergone neuroimaging: of 50.3% of patients who were imaged 27.6% had an identifiable cause for headache including intracranial hemorrhage (28.6%), CVT (23.8%); acute ischemic PRES in 19%, stroke (14.3%) and acute sinusitis in 14.3%, respectively (Table 1). An additional 14.2% of cases had incidental findings that were incidental (2).

## Discussion

### Worrying symptoms

Progressive and severe, thunderclap headache (a headache which becomes severe over a period of seconds to minutes) as well high BP during pregnancy are considered red flags (5, 6). New onset headache during pregnancy and in particular the third trimester or postpartum, lack of pregestational history of headache, pregestational history of hypertension should raise suspicion for a secondary headache. Fever, nausea and vomiting, focal neurological signs and symptoms including alteration in mental status, visual changes and seizures are also concerning (Table 2). Each of these symptoms can be associated with one or more possible diagnoses and warrant an appropriate work up (Table 2).

**Table 2:** Headache red flags in pregnancy and the puerperium.

Signs and symptomss	Differential diagnosis	Work up
“Thunderclap” headache*	Aneurysmal SAH, RCVS, PRES, cerebral artery dissection, CVT, PA	Neuroimaging, LP, blood test, endocrine tests & visual testing
Focal neurological signs	ICH, stroke, CVT, intracranial mass lesion	Neuroimaging
Elevated blood pressure	Preeclampsia, RCVS, PRES, HELLP	Blood test**, urine test***, Neuroimaging
Fever	CNS infection, sinusitis	Blood test, Neuroimaging, LP
Gestational age 3^rd^ trimester	preeclampsia, ICH, CVT, PRES	Blood test**, urine test***, Neuroimaging
Increased LFTs, ↑/↓ platelets, ↑ CRP	Preeclampsia, CVT	Blood test**, urine test***, Neuroimaging (CVT)
New onset of severe headache or worsening of previous headache in pregnancy or postpartum	CVT, carotid artery dissection, ICH, RCVS, PRES, PA, space occupying lesion	Neuroimaging
Papilledema	CVT, IIH, intracranial mass lesion, meningitis	Neuroimaging, LP, visual testing (eg, HVF if IIH is suspected)
Seizure	Eclampsia, ICH, PRES, RCVS, CNS infection, stroke, intracranial mass lesion	Neuroimaging, blood test

ICH, intracranial hemorrhage; SAH, subarachnoid hemorrhage; RCVS, reversible cerebral vasoconstriction syndrome; PRES, posterior reversible encephalopathy syndrome; PA, pituitary apoplexy; HELLP, hemolysis, elevated liver enzymes, and low platelet count; IIH, idiopathic intracranial hypertension; LP, lumbar puncture; LFTs, liver function tests; CRP, C-reactive protein; HVF, Humphrey visual field.

* Thunderclap headache is defined as a severe headache of sudden onset.

** Blood tests for evaluation of preeclampsia must include complete blood count, LFTs, blood urea nitrogen and creatinine.

*** Urine test for evaluation of preeclampsia must include urine total protein to creatinine ratio (ratio > 0.3 is consistent with preeclampsia).

### Specific causes of secondary headache in pregnancy and the puerperium

The risk of hypertension and cerebrovascular disease are increased during the pregnancy and puerperium because of a 40% increase in plasma volume, 15%–25% increase in heart rate and cardiac output, a 40% increase in vasodilation. In addition although there is an initial decrease in both systolic and diastolic blood pressure (BP) from preconception values until late second trimester (gestational age 20 to 32 weeks), BP then gradually rises until term (7).

### Preeclampsia and eclampsia

Preeclampsia affects 3%–5% of pregnancies (8). The incidence of eclampsia in the U.S. between 2009 and 2017 has been reported to be 0.3% (9). Progressive bilateral throbbing headache aggravated by exertion and not responding to over-the-counter medications may be a symptom of preeclampsia. In addition, visual changes similar to migraine visual aura may be present. Preeclampsia should be included in the differential in patients with a headache who are at least 20 0/7 weeks of gestation or within 6 weeks postpartum and who have BP elevations ≥140 systolic or ≥ 90 diastolic. Headache attributable to preeclampsia must resolve within a week after BP treatment and normalization per criteria of the International Headache Society (IHS) (10).

Migraine is also an independent risk factor for developing gestational hypertension (OR 1.2–1.68) and preeclampsia (OR range 1.08–3.5) (11). In a retrospective case control study, Bushnell et al. found cerebrovascular complications of pregnancy in 0.2% of migraine sufferers as compared to non-migraineurs (12). Conversely, migraine history is more common among women with preeclampsia, as compared to the general population with percentages ranging from 16% to 36% (13). Common pathophysiological mechanisms may be shared between migraine and preeclampsia, including alteration in vasoreactivity, endothelial and platelet dysfunction as well as decrease prostacyclin production (13). ACOG recommends treatment with magnesium sulfate and blood pressure control and delivery in women who present with persistent headache attributed to preeclampsia with severe features (14).

### Posterior reversible encephalopathy syndrome

In pregnant women, PRES is part of eclampsia/preeclampsia therefore patients with PRES should be evaluated for preeclampsia given the potential life-threatening complications for the mother and fetus. PRES is associated with vasogenic edema presumably due to the failure of autoregulation within the cerebral vasculature in the setting of increased hydrostatic pressure and endothelial cell dysfunction (15). It has been speculated that PRES predominantly affects the occipital lobes because of the relatively poor adrenergic innervation in the posterior circulation territory (16). In addition to headache, patients may also report visual symptoms, such as blurred vision or hemianopsia. It should be noted that because BP is typically lower in pregnant women as compared to non-pregnant women, BP elevations in the range typically seen in other patient populations with PRES may not be seen in pregnant women especially in the second or third trimester because of decreased vascular resistance (17).

### Reversible cerebral vasoconstriction syndrome

RCVS shares common pathophysiological mechanisms with PRES. 90% of patients with RCVS have a good outcome but complications occur in 10% including ischemic and hemorrhagic strokes, subarachnoid hemorrhage and intraparenchymal hemorrhage (18). Risk of RCVS is 3 to 9 times higher in pregnant women as compared to the general population (19).

### Intracerebral hemorrhage

In addition to vasoactive changes, pregnancy is a hypercoagulable state characterized by an increase in the level of pro-thrombotic factors and a decrease in anticoagulant factors such as protein S. ICH which can occur in the setting of stroke, rupture of an aneurysm or vascular malformation, or be due to hypertension, PRES, or RCVS, is associated with a mortality rate of around 20% (20). Therefore, its recognition and prompt treatment are critical. Thunderclap headache or the worst headache of life is a hallmark of ICH.

As to ICH associated with AVMs and cerebral aneurysms, older studies (21, 22) suggest no change in the rates of ICH during pregnancy whereas more recent studies show that the second and third trimesters may be associated with higher risk of hemorrhagic stroke due to AMVs and that the third trimester in particular is associated with higher aneurysmal rupture (23–25). There is however inconclusive evidence regarding the risk of rupture of a known cerebral aneurysms or AVM in pregnancy compared to non-pregnant state (20).

Hemorrhagic stroke also presents a thunderclap headache with or without neurological symptoms. Hemorrhagic stroke occurs with an increased frequency during pregnancy (26, 27). In a study comparing rates of hemorrhagic stroke in young women, aged 18 to 45, 14.6% of the events occur during pregnancy or the puerperium (28). An urgent magnetic resonance angiogram (MRA) or computed tomography angiography (CTA) can identify any potential vascular abnormality and prompt surgical or endovascular treatment must be pursued (20). Medical therapies include management of BP, avoidance of anticoagulant medication, and treatment in an intensive care unit.

### Acute ischemic stroke

Headache occurs in up to one third of patients with posterior circulation stroke, but it is typically associated with focal neurological symptoms and at times alteration in mental status. Headache is rarely the presenting or the predominant symptom of a stroke. Headache in the setting of stroke does not have any specific features but it appears to be somewhat more common in younger women (29–31). Pregnancy itself is not associated with an increased risk of ischemic stroke but the risk of ischemic stroke has been reported to be 3 to 9 times higher during the third trimester and the puerperium than in non-pregnant women (19). In a review in 2005, stroke was estimated to occur in approximately 30/100,000 deliveries (32). Twelve percent of all strokes in pregnancy are fatal and pose a risk to the fetus as well (33).

In a retrospective study conducted in France, 1,261 of 6,000,297, 698 pregnancies, the incidence rates for ischemic stroke were similar in pregnant and nonpregnant women (34). Risk factors associated with stroke in pregnancy included history of tobacco usage, pre-existing HTN, preeclampsia and eclampsia, diabetes, and gestational diabetes (34). Other studies also report that preeclampsia and eclampsia are the strongest risk factors for stroke (35, 36).

Imaging of stroke in pregnancy is similar to nonpregnant women (37). According to ACOG clinical guidelines, CT is the test of choice in pregnant women suspected of having a stroke (14). MRI (magnetic resonance imaging) time-of-flight and MRA are preferrable to CTA. Because of reports of uterine hemorrhage associated with its usage, there are no clear guidelines for the use of intravenous alteplase in pregnancy and each patient should be managed on a case-to-case basis once the risk based on the extent of the stroke and its potential benefit have been estimated.

### Cerebral venous thrombosis

CVT occurs in one per 2,500–10,000 pregnancies and mostly in the third trimester, postpartum or peripartum period (38). As compared to the general population where the CVT causes about 0.5%–1% of all strokes, the incidence of CVT associated stroke in pregnancy is 2% and its prevalence is about 12/100,000 pregnancies (39). Headache was the most common presenting symptom during pregnancy in 86.1% and during the puerperium in 18.3% of CVT patients in a large retrospective study (40). HA tends to be paroxysmal in onset, severe and throbbing. It can be holocephalic or unilateral (41) and have migraine like features (42). In addition, focal neurological symptoms, seizures, blurred vision as well as nausea and vomiting may be present (43). When headache is the only presenting symptom, CVT has been shown to be associated with a better outcome whereas alteration in mental status is associated with a worse outcome (39). For treatment of CVT adjusted dose low molecular weight heparin is recommended to prevent long-term sequela (14).

### Brain tumors

Headache is a common presenting feature of brain tumor along with focal neurological complaints and symptoms of increased intracranial pressure such as nocturnal headache, nausea, vomiting and blurred vision. Tumors such as pituitary adenomas and meningiomas may grow during pregnancy (44).

### Meningiomas

Although there is no absolute increase in the incidence of meningiomas during pregnancy (45), evidence suggests growth of meningiomas and a dramatic onset of symptoms due to previously asymptomatic meningiomas during pregnancy, as well as shrinkage of the tumor after delivery (46, 47). A strong association exists however between the female gender, female sex hormone levels, and meningiomas (47). Meningiomas have progesterone receptors (48). Meningiomas in pregnancy are typically diagnosed during the second and third trimester.

In one large review of pregnant women with newly diagnosed brain tumors, about 40% of pregnancy related intracranial meningiomas were surgically resected during pregnancy and about 60% were surgically excised after giving birth. No statistical differences were found between the two groups in terms of tumor related variables and clinical outcome (49).

### Pituitary adenomas and pituitary apoplexy

During normal pregnancy, the pituitary gland volume increases up to 120% of its original size due to hyperplasia of the prolactin cells, and the metabolism of the pituitary gland changes significantly as a result of placental hormone secretion (50, 51).

Headache and visual disturbance usually occur when the size of the tumor increases to more than one centimeter. Pituitary apoplexy (PA) which refers to hemorrhage or infarction of the pituitary gland is the most ominous consequence of a pituitary adenoma or a physiologically enlarged pituitary gland (52). In addition to acute headache, PA is accompanied by visual impairment and pituitary dysfunction. Early diagnosis is important because if untreated, PA can lead to a neuroendocrine emergency with acute central hypoadrenalism, hyponatremia and hypotension (53). Women with known pituitary adenomas require neurologic and ophthalmic evaluation every 3 months during pregnancy (52). Imaging is recommended only if there is evidence of tumor growth on symptomatic grounds. The preferred mode of pituitary imaging in pregnancy is non-contrast MRI (37).

Pregnancy associated tumors such as choriocarcinomas may rarely metastasize to the brain during pregnancy. Choriocarcinoma occurs in about 1 in 40,000 pregnancies (54). If choriocarcinoma metastasizes to the brain it is associated with headache, and other symptoms of increased intracranial pressure. For diagnosis brain MRI is preferred. If brain MRI is negative a lumbar puncture is recommended to determine the level of chorionic gonadotropin HCG in the spinal fluid. A ratio of HCG of <40:1 blood compared to CSF is consistent with CNS metastasis (55).

Lesions such as colloid cysts or Chiari malformations can also present with headache during childbirth due to Valsalva forces.

### Postdural puncture headache (PDP)

Intracranial hypotension is a well-known complication of postdural puncture in the postpartum period. Headache in PDP is orthostatic in nature, i.e., it improves in the recumbent position and worsens by standing up. Headaches are usually severe, associated with dizziness and nausea. In a retrospective study of acute headache in the postpartum, PDP accounted for 45.7% of all secondary headaches (56). Rare instances of spontaneous intracranial hypotension have also been reported (57). Epidural blood patch is the treatment of choice for patients with PDP (14).

### Other causes of secondary headache during pregnancy

One of the most common causes of headache in the postpartum period is due to musculoskeletal pain which is not considered a SH. This results from maternal physical exertion during labor and associated sleep deprivation. This headache type is associated with neck and shoulder pain, and no history of dural puncture. Rare instances of cerebral artery dissection, idiopathic intracranial hypertension resulting from weight gain, sinusitis ad meningitis have also been reported as cause of headache during pregnancy, but their presentation is not different in these instances from patients who are not pregnant and pregnancy is not associated with increased risk for these diagnoses.

### Imaging in pregnancy

Timely imaging is key and the use of brain imaging is required in at risk patients.

MRI is the preferred neuroimaging modality in pregnant women because it does not involve the use of ionizing radiation and allows for better soft tissue evaluation as compared to CT (58). There may be however some concern about obtaining an MRI in the first trimester because of the possible exposure of the fetus to the heat generated by the magnetic field and the strong noise exposure (59, 60).

Use of contrast, such as gadolinium should be avoided because of potential lack of safety to the fetus given its ability to cross the placenta and to accumulate in the amniotic fluid unless its use significantly improves diagnosis (37, 43, 59). MRI brain with time-of-flight is preferrable to CTA in cases that warrant cerebrovascular imaging except in cases where urgent diagnostic information is needed should MRI not be immediately available. Data collected on children born to women who had undergone contrast enhanced MRI during pregnancy does not show an increase in the rate of congenital malformation but its use may be associated with higher likelihood of stillbirth and neonatal death as well as rheumatological and inflammatory disorders (60). If stroke is suspected, CT is the modality of choice because the ionizing radiation dose from a single head CT which exposes the fetus to a dose of 0.001–0.01 mGy does not pose a risk (37) but in other scenarios, MRI, MR Venogram and MRA without contrast are preferentially recommended (61, 62).

## Conclusion

Physiological and endocrine changes occurring during pregnancy increase the risk of secondary headache disorders. New onset headache especially in the third trimester or postpartum, no prior history of headache, clinical signs of preeclampsia/eclampsia, and pregestational hypertension are additional red flags that need to be considered in patients presenting with headache during pregnancy or the puerperium in addition to other headache features such as thunderclap headache and focal neurological signs and symptoms. Timely imaging is key because in more than 25% of pregnant women presenting with acute headache brain imaging may reveal a secondary etiology (1, 2). MRI is the preferred imaging modality in most cases.

## Author contributions

The author confirms being the sole contributor of this work and has approved it for publication.
